# The Temperature of Workrooms

**Published:** 1903-01-31

**Authors:** 


### The Temperature of Workrooms.

It is hardly to be expected that the change of opinion which has come about in regard to the sanitary excellencies of warmth, and the popularisation of knowledge as to the virtues of cold air which has resulted from the "crusade against consumption," will remain for long without influence upon current ideas as to the temperature at which workrooms should be maintained. The heavy fines lately inflicted upon certain Court dressmakers in the West End for not securing and maintaining a reasonable temperature in rooms in which women were employed, in such a manner as not to interfere with the purity of the air, gives point and urgency to a matter which affects the workers in many trades. It is to be noted that the demands of the Factory and Workshops Act, in regard to this matter, may be considered from two points of view—that of temperature and that of purity—and it is but fair to believe that, in inflicting the penalties which he did, the magistrate had regard quite as much, if not more, to the evidence which was given as to the "gaseous, fœted, bad, and injurious" quality of the atmosphere in the workrooms, than to the fact that the temperature was only 58° in the one case, and between 54° and 56° in the other. Evidence was given in the one case that the room in which the women had to work was mainly heated by ordinary gas jets, supplemented by a gas stove used for heating flat-irons, and that although the windows were opened at the top a short space, the atmosphere was vitiated and quite unhealthy to live in; and in the other case, that there was no heating apparatus beyond a small iron heater in the grate and four gas jets. What is a reasonable temperature must obviously depend to a large extent upon the nature of the work done, and upon the extent to which it is possible for the workers to be thickly clad without interfering with their occupation; but in view of what is now known as to the health-giving properties of cold air, we doubt whether, as a matter of science, it could be maintained that a comparatively low temperature in workrooms was unhealthy, granting always that the operatives were well clad. Indeed, a great deal of evidence could be obtained from northern manufacturing districts to show that on the average far better health is enjoyed by woollen workers, who work in the cold, compared with cotton operatives, who work in heated rooms; while, of course, the evidence derived from modern experience of sanatorium treatment goes entirely in favour of the healthiness of cold air. As a matter of fact, the evils arising from cold workrooms largely depend, first, upon the inefficient manner in which most of the operatives are clothed; and, secondly (indeed, one might almost say consequently), upon the ineradicable tendency of ill-clad and ill-fed people to keep warm at all costs, and to put up with any amount of foulness of air rather than suffer the slightest suspicion of chill. There was much truth in the remark made by the old lady who said that it took years of good feeding to make people fond of open windows, and it is to be feared that at the present day the only way in which people can be induced to put up with ventilation is to give along with it a compensating amount of warmth. Lest, however, the public should, as is their habit, put the cart before the horse, it is worth while insisting upon the fact that, except for the increased ventilation thereby rendered practicable, the warmth so given by no means adds to the healthiness of the air, notwithstanding all that Acts of Parliament may suggest to the contrary.

